# A Lightweight Approach to Comprehensive Fabric Anomaly Detection Modeling

**DOI:** 10.3390/s25072038

**Published:** 2025-03-25

**Authors:** Shuqin Cui, Weihong Liu, Min Li

**Affiliations:** School of Computer and Artificial Intelligence, Wuhan Textile University, Wuhan 430072, China; csq@wtu.edu.cn (S.C.); 2008031@wtu.edu.cn (M.L.)

**Keywords:** lightweight network, fabric anomaly detection, lamp pruning technique, knowledge distillation

## Abstract

In order to solve the problem of high computational resource consumption in fabric anomaly detection, we propose a lightweight network, GH-YOLOx, which integrates ghost convolutions and hierarchical GHNetV2 backbone together to capture both local and global anomaly features. At the same time, other innovative components, such as GhostConv, dynamic convolutions, feature fusion modules, and a shared group convolution head, are applied to effectively handle multi-scale issues. Lamp pruning accelerates inference, while channel-wise knowledge distillation enhances the pruned model’s accuracy. Experiments on fabric datasets demonstrate that GH-YOLOx can effectively reduce the number of parameters while achieving a higher detection rate than other lightweight models. Overall, our solution offers a practical approach to real-time fabric anomaly detection on mobile and embedded devices.

## 1. Introduction

In the textile industry, fabric anomaly detection is a very important procedure to control the quality of the product [1]. The fabric anomaly inspection process can be carried out either by human inspectors or via automated fabric inspection machines. Compared to human inspection, automated fabric inspection is more reliable, objective, and stable [2]. Since the 1980s, researchers have been studying the automatic fabric anomaly detection method based on image processing and pattern recognition [3]. Recently, motivated by the development of deep learning technologies, researchers have applied deep learning algorithms, particularly convolutional neural networks (CNNs) to detect the anomaly for fabrics [4], and the detection accuracy of the deep learning-based methods is much higher than that of the image processing-based methods.

Although the deep learning-based methods are more effective than the traditional automatic method, they also face many challenges since the background of the fabric image is complex, the category of a defect is large, the shape and structure of the defect are random, and the contrast between the background and the defect is low. Furthermore, the parameters of the deep learning model are huge; therefore, the deep learning-based anomaly detection methods have high computational complexity and require large storage and computation resources, which constrains their deployment on mobile and embedded devices.

To address these challenges, by integrating various lightweighting techniques together, such as dynamic convolution, lightweight shared convolutional heads (LSCDs), layer-wise automatic magnitude pruning (LAMP), and channel-wise knowledge distillation (CWD). This paper develops a lightweight network framework, GH-YOLOx, for fabric anomaly detection. By integrating these lightweight strategies, GH-YOLOx exhibits exceptional performance in fabric anomaly detection and offers a robust solution for practical applications in this domain. Furthermore, by embedding the backbone network, neck network, and LSCD of GH-YOLOx into YOLOv5 and YOLOv8, experiments were conducted on the public fabric dataset [5]. The experimental results demonstrate that these modules significantly reduce the computational load of the network while enhancing detection speed. Additionally, to evaluate the generalization capability of the model, GH-YOLO after pruning and knowledge distillation was compared with other mainstream models on both a public fabric dataset [5] and the NEU surface dataset [6]. The findings reveal that its performance surpasses that of other lightweight networks. In summary, our method achieves the following key contributions.

(1) Lightweight Network GH-YOLOx.

The network uses an HGNet backbone with GhostNetV2 lightweight convolutions and a “split–transform–concatenate” strategy to significantly reduce parameters, while dynamic kernel adjustments enhance the detection of small defects; an improved C2f module (with GhostConv, dynamic convolution, and skip connections), along with a shared-weights group convolution detection head, achieves efficient multi-scale feature fusion and precise detection.

(2) Lightweight collaborative scheme.

A LAMP-based pruning strategy removes redundant channels layer by layer, preserving key features; coupled with channel-wise distillation (CWD) that leverages a teacher model to transfer attention and restore the pruned model’s capacity, it strikes a balance between efficiency and accuracy.

## 2. Related Work

Traditional fabric defect detection has long relied on manual inspection (Wang et al., 2018 [7]) or classical image processing algorithms. For example, Chen et al., 2023 [8], proposed a multi-scale texture analysis method based on Gabor filtering, which improved defect recognition accuracy to 82% for plain fabrics but suffered from 18% false detection rates for complex jacquard fabrics. In 2021, Liu et al. [9] developed a threshold-adaptive algorithm based on morphological segmentation, reducing the missed detection rate to 12% under uniform lighting but failing to handle local contrast variations caused by shadows. Sun et al., 2022 [10], fused frequency-domain features with sparse optimization strategies to reconstruct image residuals for defect localization, achieving a 10% accuracy improvement over traditional methods, yet increasing computational latency to 200 ms per frame. Wang et al., 2023 [11], proposed an improved visual saliency model using the Histogram of Oriented Gradients (HOG) to enhance small target detection, but segmentation performance remained suboptimal in high-density overlapping defect regions. Li et al., 2022 [12], designed a texture distortion detection algorithm based on monogenic wavelet analysis, achieving 85% recall in warp-knitted fabrics but requiring manually set feature extraction parameters, limiting generalization.

**Figure 1:**
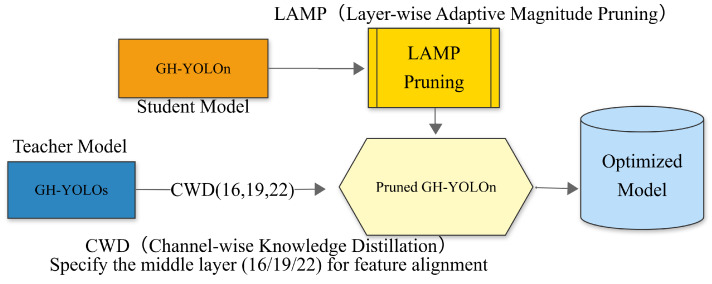
Integrated framework process.

To address these limitations, deep learning models have emerged as a research focus: Liu et al., 2021 [13] constructed a cascaded U-Net achieving 89% classification accuracy through multi-level feature fusion, but its 230M parameters hindered deployment on embedded devices. Xu et al., 2021 [14] proposed a dual channel-spatial attention module to improve mAP to 78.5%, yet inefficient multi-scale fusion limited the inference speed to 15 FPS. Zhao et al., 2023 [15], replaced ResNet with MobileNetV3 as the backbone, reducing the model size by 40% but increasing missed detection rates for sub-5px defects by 9%. Huang et al., 2023 [16], designed a detection-segmentation joint framework for pixel-level localization (mAP 82.3%), but they required model compression below 30M for Jetson TX2 compatibility. Wang et al., 2023 [17], improved RefineDet with dynamic label assignment, boosting mAP by 6.2% in complex texture scenarios, yet regression errors persisted for defects with extreme aspect ratios. These methods struggled to meet real-time requirements and adapt to embedded resource constraints. Consequently, lightweight technologies have been explored. Backbone lightweighting: Zhang et al., 2024 [18], replaced CSPDarkNet53 in YOLOv4 with GhostNet, reducing parameters by 83.5% but limiting cross-scene generalization. Chen et al., 2023 [19], proposed FasterNet, lowering the computational cost to 38.6 GFLOPS but retaining 5.5% missed detection rates for high-density defects. Neck lightweighting: Li et al., 2024 [20], designed dynamic convolution modules with feature pyramid weighting, improving small target accuracy by 2.75% at the cost of a 25% hardware overhead. Huang et al., 2024 [21], enhanced C2f modules with channel attention (CA), increasing mAP by 4.6% but failing to optimize multi-scale fusion efficiency. Head lightweighting: Kim et al., 2023 [22], proposed shared convolutional heads to reduce parameters by 15%, yet they introduced 3% localization errors for irregular defects. Liu et al., 2024 [23], employed group convolution to compress head parameters, boosting the inference speed by 20% but requiring complex fine-tuning to recover accuracy. However, these methods focused on local structural optimization with limited global compression. Thus, researchers further explored pruning and knowledge distillation. Pruning: Zhao et al., 2023 [24], introduced gradient magnitude pruning, compressing models by 50% with only a 2.1% accuracy loss, yet texture-sensitive channel degradation caused a 6% mAP drop in fabric scenarios. Quantization–distillation: Pei et al., 2023 [25], designed a hybrid quantization–distillation scheme, achieving 20 ms inference on edge devices, but adversarial training introduced noise interference. Domain-adaptive distillation: Tonis et al., 2023 [26], proposed fabric-aligned distillation (FAD) to transfer texture features from ResNet50 teachers, compensating for a 1.5% post-pruning accuracy loss, yet requiring high-quality labeled data. Therefore, in view of the unique characteristics of the high-density, small-target, and low-contrast texture of fabric defects, this paper proposes a network architecture that fuses the overall lightweight design of the trunk, neck, and detection head and combines the collaborative optimization strategy of gradient-guided pruning (LAMP) and fabric feature-aligned distillation (CWD). Through domain-customized design, the performance bottleneck of existing methods in the precision–lightweight trade-off is broken. The specific workflow is illustrated in Figure 1.

## 3. GH-YOLOx

### 3.1. Network Structure

This paper presents GH-YOLOx, a lightweight object detection model based on YOLOX. Initially, we amalgamate GhostConv, DWConv, and a lightweight backbone network, HGNetV2, to develop a novel backbone network called GH-HGV2Net, which significantly reduces the number of parameters. Subsequently, in the feature extraction phase, we integrate the feature-rich DynaMicConv with C2f and GhostConv to construct a dual-stream PANet for feature fusion, thereby enhancing the model’s ability to focus on small defects and improving feature representation. Finally, the application of LSCD detection heads further minimizes the model’s parameter count while simultaneously boosting its accuracy and inference speed.

**Figure 2:**
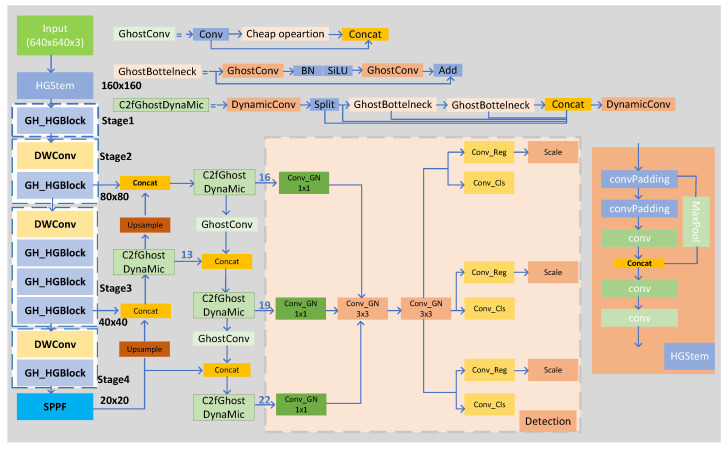
Network structure.

GH-YOLOx is composed of three main components: a backbone network, a feature extraction module, and a lightweight YOLO head. The overall architecture of the network is depicted in Figure 2. After passing through the HGStem module, four stages of GH-HGBlock, and the SPPF module, the backbone network generates three output feature maps with dimensions of (80, 80, 512), (40, 40, 1024), and (20, 20, 1024). Within the C2GD-PAN structure, the feature maps are fused in a bidirectional manner, undergoing two downsampling operations and two upsampling operations. This process yields three feature maps corresponding to small, medium, and large objects, respectively labeled as 16, 19, and 22. These feature maps are then passed to the LSCD module for prediction.

#### 3.1.1. Backbone

The hierarchical structure of the backbone network facilitates the effective handling of both local and global structures of fabric anomalies. This design enables feature extraction at different levels, thus improving the capture of both local fine structures and global overall structures in images. Based on this, we introduce GH-HGNetV2, a backbone network tailored to fabric anomaly detection. It comprises HG stages with GH-HGV2 blocks, each using GhostConv in its convolution operations. The GhostConv convolution extracts feature information from the input feature map using a small number of ordinary convolutional kernels, followed by cheaper linear transformation operations to generate the final feature map. This distinctive convolutional operation reduces network computational complexity and parameter count while preserving the original feature map dimensions and channel numbers, thereby enhancing both computational efficiency and network performance. Moreover, the backbone network includes an HGStem module, which has standard convolutional and pooling layers. This module lowers the input fabric image’s resolution to a level suitable for network processing while extracting initial feature representations, thus reducing the computational load.

The entire backbone network comprises an HGStem module and four HG stages aimed at improving the accuracy and robustness of fabric anomaly detection through hierarchical feature representation and multi-scale feature extraction. Finally, the output features of the backbone network are inputted into an SPPF (spatial pyramid pooling fusion) module to extract and fuse multi-scale feature information. Through hierarchical feature representation, hierarchical feature representation facilitates the accurate capturing of both local and global structures in fabric images, enabling the sensitive detection of subtle defects. This process further enhances the network’s detection capability and robustness.

**Figure 3:**
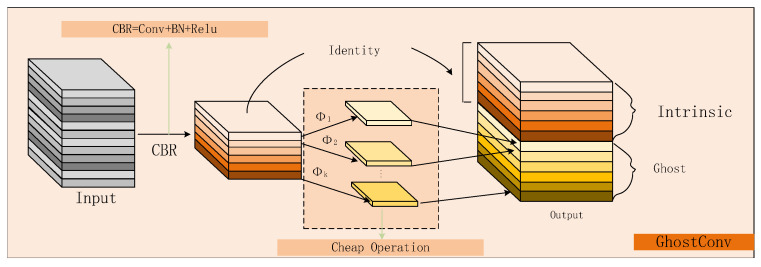
GhostConv

As shown in Figure 3, The direction of the data arrow represents the direction of the feature map flow. the ghost convolution process is divided into two stages. In the first stage, a small number of convolutions are applied (for example, instead of using 128 convolution kernels as usual, here, only 64 are used, reducing the computational load by half). In the second stage, known as the cheap operation stage, denoted as Φ in Figure 3, operations like 3 × 3 or 5 × 5 convolutions are performed individually on each feature map (depth-wise convolutional). The convolution operation comprises a convolution–batch normalization (BN)–non-linear activation combination, while the term “cheap operation” refers to regular convolution without batch normalization or non-linear activation. Final feature maps are generated through concatenation of outputs from both stages, preserving the original channel dimension while maintaining computational efficiency.

**Figure 4:**
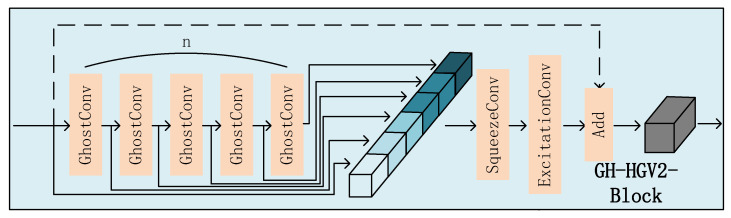
GH-HGV2Block

As illustrated in Figure 4, The direction of the data arrow represents the direction of the feature map flow. the GH-HGV2 block serves as the fundamental building unit of the backbone network. Its internal process is as follows: Firstly, the input features undergo n GhostConv convolution operations, generating a series of feature maps. Subsequently, these feature maps are concatenated through the concatenate operation, combining the feature maps produced by each GhostConv operation. Next, the concatenated feature maps undergo SqueezeConv and ExcitationConv operations to extract channel information and feature responses. Finally, if shortcut or C1=C2 holds, the original input and the maps from GhostConv and Concat are summed for the final feature map. This design fully leverages the advantages of GhostConv convolutional operations to reduce network computational and parameter overheads. Additionally, by incorporating the concatenate and summation operations, it merges feature information from different stages, thereby enhancing the network’s feature representation capability and detection performance.

#### 3.1.2. Neck

Fabric anomalies can be minor, sometimes just a few pixels. Given that these defects may resemble the fabric’s texture, it is essential to enhance the detection accuracy of subtle defects when designing lightweight neck networks. This requires more sensitive feature extraction methods to distinguish between slight differences in defects and normal textures. Incorporating dynamic convolution into the feature network is an excellent choice. Dynamic convolution offers stronger feature representation capabilities, enabling better discrimination between fine textures and defects and thus contributing to the improved detection accuracy of the model. Although dynamic convolution may introduce some computational overhead, the benefits it brings in terms of accuracy improvement are usually worthwhile. The newly designed module C2GD-PAFPN (Neck) is a refinement of the C2f module, where the ordinary convolution blocks inside the bottleneck are replaced with GhostConv and dynamic convolution. This structure utilizes a cross-stage feature fusion strategy and gradient truncation flow technique to enhance the variability of feature learning between different network layers, thereby reducing the impact of redundant gradient information and strengthening the network’s learning capability. With the introduction of GhostConv and dynamic convolution, the C2GD-PAFPN module reduces a significant number of 3 × 3 ordinary convolutions in the original structure, greatly compressing the model size, reducing the parameter count and computational load, enabling the model to be deployed on mobile devices, and facilitating edge computing-based defect detection in fabrics. The specific architecture is illustrated in Figure 1 below.

The backbone network extracts 20 × 20 × 3 features, which are first processed via the SPPF (spatial pyramid pooling fusion) module. Subsequently, an upsampling operation is carried out, and at the same time, the downsampled feature map is concatenated with the 40 × 40 × 3 feature map provided via the third stage. After that, the concatenated features pass through a C2f module (labeled layer 13), and then another upsampling operation is performed. The upsampled feature map is then concatenated with the 80 × 80 × 3 feature map provided via the second stage. Next, the features go through another C2f module (labeled layer 16), followed by a GhostConv upsampling operation, generating features used to detect small targets. Afterward, the upsampled feature map is concatenated with the initially concatenated features (layer 13), and then it passes through another C2f module (labeled as layer 19), providing features for detecting medium-sized targets. Additionally, the features from layer 19 undergo another GhostConv operation and are then concatenated with the 20 × 20 × 3 features processed via the SPPF. Finally, the concatenated feature map passes through another C2f module, providing features for detecting large targets. Thus, the flow of feature maps in the feature fusion network is completed.

#### 3.1.3. Head

To address the challenges of reducing model computation and accommodating the diverse sizes and shapes of fabric defects, this paper proposes an innovative detection head named LSCD (layer-wise scale and convolution-decoupled detection head). LSCD adopts a grouped shared-weight strategy, using the same set of convolutional kernels to predict detection boxes across multiple scales of feature maps from the FPN (feature pyramid network). Subsequently, each layer utilizes a learnable scale factor as a coefficient to scale the predicted boxes. GroupNorm, validated in [x], has been shown to enhance the performance of detection head localization and classification. By incorporating shared convolutions, LSCD drastically reduces parameters, making the model lighter and suitable for resource-constrained devices. In the shared convolutional layers, all normalization layers are replaced with GroupNorm. The rationale for sharing parameters in the classification loss is that the learned information in the detection layer is consistent, regardless of the target size. However, for regression loss parameter sharing, scale must be considered. Hence, a scale layer is introduced to the scale features, addressing scale inconsistency across detection heads.

The specific process is illustrated in Figure 2, operating on the features extracted at layers 16, 19, and 22 of the network. Specifically, these features are first processed through Conv-GN1x1 (grouped convolution) to reduce computational complexity. Subsequently, the features processed via Conv-GN1x1 are inputted into two Conv-GN3x3 grouped convolutions with shared parameters to further extract feature information. After the grouped convolution with shared parameters, the features are divided into three branches for detecting objects of different sizes, including small, medium, and large objects. Within each branch, shared parameter convolutions, Conv-Reg, and Conv-Cls, are employed again for target regression and classification tasks, respectively. It is worth noting that, in each Conv-Reg operation, we introduce a trainable scalar parameter called “scale” to adjust the scale of target regression, thereby accommodating different sizes of fabric defects. The introduction of the scale parameter allows our model to flexibly adapt to various sizes and shapes of fabric defects. By optimizing the scale parameter during training, the model can effectively detect and locate objects of different sizes, improving detection accuracy and robustness.

## 4. Lamp

This paper employs the layer-wise automatic magnitude pruning (lamp [14]) technique to further compress and accelerate improved lightweight networks. Compared to other pruning methods, lamp pruning requires no hyperparameters and does not rely on any specific knowledge of the model. Its principle involves treating each neural network layer as an operator, determining the priority of magnitude-based pruning (MP) for layer-wise sparsity by examining the “model-level” distortion introduced by pruning layers. This is achieved by adjusting weight magnitudes based on lamp scores to approximate the model-level distortion caused by pruning, automatically determining layer-wise sparsity and thereby achieving the further compression and acceleration of lightweight networks. Global pruning with lamp scores is akin to MP with automatically determined layer-wise sparsity. Moreover, utilizing lamp pruning preserves the advantages of MP.

The optimization problem is represented as shown in Equation (1):

(1) $$\min_{\sum_{i=1}^{d}{∥M^{\left(i\right)}∥}_{0}\leq \kappa }\underset{{∥x∥}_{2}\leq 1}{\sup}{∥f\left(x;W^{\left(1:d\right)}\right)-f\left(x;{\tilde{W}}^{\left(1:d\right)}\right)∥}_{2}$$

where κ denotes the model-level sparsity constraint. Under the greedy strategy, the connection with the lowest importance score is removed every iteration. According to the definition of the spectral norm and the condition of $∥A∥\leq {∥A∥}_{F}$, the above optimization can be relaxed using the following upper bound of the model output distortion, as shown in Equation (2):

(2) $$\begin{array}{cc} \; & \underset{{∥x∥}_{2}\leq 1}{\sup}{∥f\left(x;W^{\left(1:d\right)}\right)-f\left(x;W^{\left(1:i-1\right)},{\tilde{W}}^{\left(i\right)},W^{\left(i+1:d\right)}\right)∥}_{2}\\ \; & \leq \frac{∥W^{\left(i\right)}-{\tilde{W}}^{\left(i\right)}{∥}_{F}}{∥W^{\left(i\right)}{∥}_{F}}\left(\prod_{j=1}^{d}{∥W^{\left(j\right)}∥}_{F}\right) \end{array}$$

Flatten the weight tensor $W^{\left(i\right)}$ to a one-dimensional vector and sort it to be satisfied whenever u<v. Since the product term |W[u]|≤|W[v]|$\prod_{j=1}^{d}{∥W^{\left(j\right)}∥}_{F}$ is a constant value, the importance score for the *u*-th index of the weight tensor *W* is defined as follows: the lamp score for the *u*-th index of the weight tensor W is then defined as

(3) $$\mathrm{score}\left(u;W\right):=\frac{{\left(W\left[u\right]\right)}^{2}}{\sum_{v\geq u}{\left(W\left[v\right]\right)}^{2}}$$

In simple terms, the lamp score (Equation (3)) quantifies the relative importance of the target connection among all surviving connections within the same layer, where connections with smaller weight magnitudes (within the same layer) have already been pruned. Consequently, two connections with identical weight magnitudes have different lamp scores, depending on the index mapping used. Once the lamp scores are computed, we globally prune the connections with the smallest lamp scores until the desired global sparsity constraint is met. This process is equivalent to performing magnitude pruning (MP) with automatically selected layerwise sparsity.

## 5. Channel-Wise Knowledge Distillation

To enhance the pruned model, knowledge distillation emerges as an effective approach to improving algorithm detection accuracy. Existing knowledge distillation methods typically employ point-wise alignment or structured information alignment between spatial positions. However, channels contain a vast amount of knowledge that often gets overlooked during distillation. Channel-wise knowledge distillation [15] can better utilize the knowledge within each channel, with activations between teacher and student networks corresponding to respective channels being adjusted gradually. To achieve this, activations of channels are first transformed into probability distributions, allowing the utilization of probability distance metrics such as KL divergence to measure differences. Representing teacher and student networks as *T* and *S*, and their activation mappings as $y^{T}$ and $y^{S}$, respectively, the general form of channel-wise distillation loss is expressed as shown in Equation (4):

(4) $$\varphi \left(\phi \left(y^{T}\right),\phi \left(y^{S}\right)\right)=\varphi \left(\phi \left(y_{c}^{T}\right),\phi \left(y_{c}^{S}\right)\right)$$

To convert activation values into probability distributions, ϕ(·) is used as shown in Equation (5):

(5) $$\phi \left(y_{c}\right)=\frac{\exp\left(\frac{y_{c,i}}{\tau }\right)}{\sum_{i=1}^{W\times H}\exp\left(\frac{y_{c,i}}{\tau }\right)}$$

where c=1,2,…,c indexes the channels, and i indexes the spatial positions of the channels. τ is a hyperparameter (temperature). If we use a larger τ, the probabilities become softer, meaning we focus on a broader spatial region for each channel. By applying softmax normalization, we eliminate the scale influence between large and compact networks. If the number of channels between the teacher and student networks does not match, 1 × 1 convolutional layers are used to upsample the channels in the student network. The lightweight network framework GH-YOLOx proposed in this paper consists of three scales of networks: n, s, and m. In this paper, the m model is used as the teacher model, and the n model is used as the student model. Specifically, knowledge distillation is performed on three feature layers (small, medium, and large) (16, 19, 22) extracted from the backbone network, as shown in Figure 1.

## 6. Experiment and Result Analysis

The model was tested on a dataset of fabric- [5] and steel-defect images. Comparative experiments were conducted against established object detection architectures including YOLOv5, YOLOv5-Lite, YOLOv3, Tiny-YOLOv3, YOLOv8, NanoDet, and Faster R-CNN under identical hardware configurations. The experimental results demonstrate that our model achieves superior detection accuracy compared to Tiny-YOLO-v3. Additionally, it exhibits a significantly faster detection speed and has substantially fewer model parameters than the other networks under investigation.

### 6.1. Dataset

**Figure 5:**
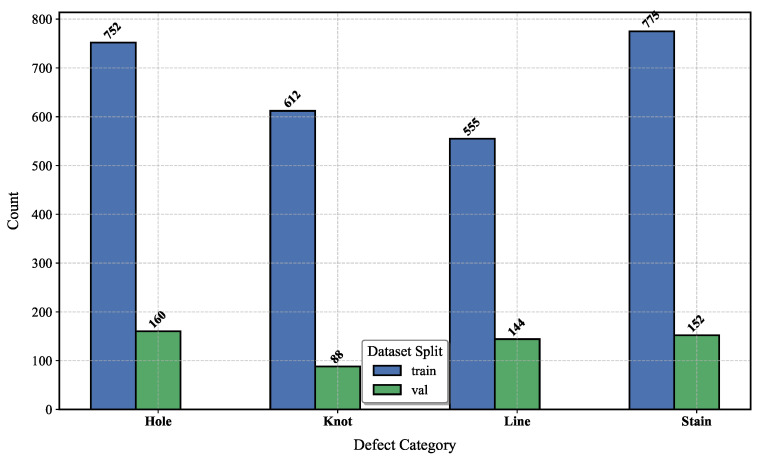
The distribution of defect categories in the fabric-defect dataset.

**Figure 6:**
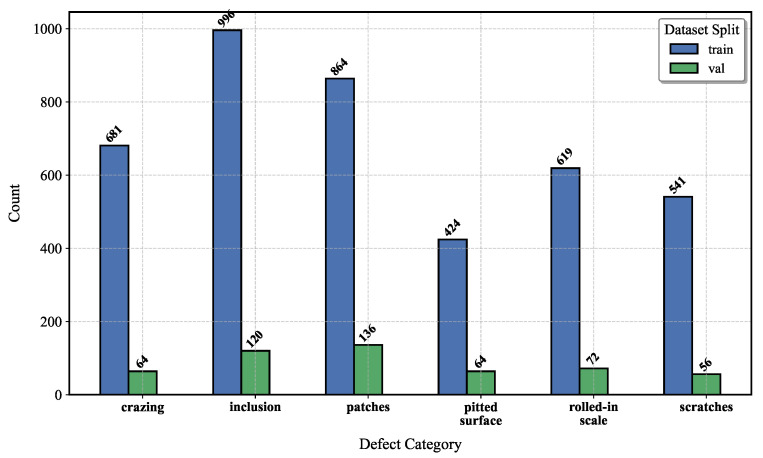
The distribution of defect categories in the steel-defect dataset.

**Figure 7:**
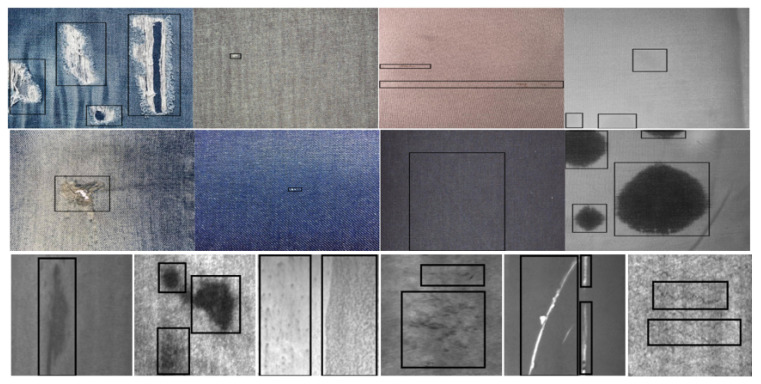
Part of the dataset for fabric defects and steel defects.

Data1 and Data2 [5,6] are both publicly available datasets. Data1 consists of 2405 fabric defect images, covering four categories of defects (Hole, Knot, Line, and Stain), as Figure 5 shows. Data2 comprises 1800 images of steel defects, encompassing six categories (Crazing, Inclusion, Patches, Pitted-Surface, Rolled-in-Scale, and Scratches),as Figure 6 shows. Part of the fabric defect images is displayed in Figure 7. The first two rows represent the fabric dataset, while the third row represents the steel dataset, with categories listed from left to right, as mentioned above. In this experiment, data augmentation was performed to double the size of the datasets; 80% of the augmented data were allocated to training and the remaining 20% to testing.

### 6.2. Training Parameters

The input size of the image is 640 × 640. The experiment was conducted using the Ubuntu 20.04.4 operating system. The CPU model was the Intel i9-9900k@3.60GHz, and the GPU model was the NVIDIA GeForce GTX3060Ti graphics card with 8G video memory with python-3.8.0, cuda-11.7, and pytorch-2.0.1.

### 6.3. Ablation Experiment

This paper proposes GH-YOLOx, a systematically engineered lightweight framework for fabric defect detection comprising three synergistic components: (1) the Ghost-HGNetV2 backbone integrating GhostConv operations with the HGNetV2 architecture through depthwise separable convolution for enhanced parameter efficiency, (2) the C2GD-PAN neck combining DynamicConv, GhostConv, and YOLOv8’s C2f module into C2f-DG blocks within a PAN structure, and (3) the lightweight shared convolutional detection (LSCD) head employing group convolution principles. The implementation progresses through three optimization phases: initial architectural design integrating hybrid convolution operations, subsequent network compression via layer-adaptive magnitude-based pruning (LAMP), and final model refinement through channel-level knowledge distillation, collectively establishing a comprehensive lightweight deployment solution.

To verify the effectiveness of the methods in steps (1), (2), and (3), ablation experiments were conducted successively. This section first validates step (1), where the modules Ghost-HGNetV2, C2GD-PAN, and LSCD in step (1) were replaced with the corresponding modules in v5 (anchor-free) and v8, and ablation experiments were carried out on the fabric dataset. Then, step (2) was validated by pruning GH-YOLOn with pruning ratios of 1.5, 2.0, and 2.5 to find the appropriate pruning ratio. Other pruning methods were also compared. Finally, step (3) was validated by comparing different knowledge distillation methods.

As shown in Table 1, A represents the GH-HGV2 backbone network, B represents the C2GD-PAN feature fusion network, and C represents the LSCD detection head network. Initially, each module is replaced with the corresponding modules in the backbone, neck, and head networks of v5 and v8. When A, B, and C are embedded in other networks, the mAP values of the model show an improvement. For v5, the highest improvement is 1.1, and the lowest is 0.5. For v8, the highest improvement is 1.3, and the lowest is 0.4. For A, in YOLOv5, the A module reduces 0.5 M parameters and improves mAP by 0.9%; in YOLOv8, it reduces 0.9 M parameters and improves mAP by 0.8%. This indicates that A can ensure the improvement of model performance and reduce the computational load of the model. For B, the highest mAP gain in YOLOv5 suggests that replacing static convolution with dynamic mechanisms balances the computational overhead. For C, the best performance in YOLOv8 is +1.3%, proving shared group convolution suits dynamic architectures and effectively trims redundant parameters. Finally, the combined network composed of A+B+C achieves YOLOv8, which is more sensitive to module improvement and achieves an mAP improvement of 1.3% compared to 0.9% for YOLOv5, indicating that its architecture is more friendly to lightweight improvement. Both achieve a double improvement in mAP and FPS, and a decrease in Params. This shows that the proposed improvement not only achieves an overall performance improvement but also enhances the performance of individual modules or their combinations when embedded in other networks.

In this paper, different pruning methods are compared on the basis of the lightweight network GH-YOLOx, including global pruning with various techniques and lamp pruning at compression ratios of 1.5, 2.0, and 2.5 based on the computational load. Since the importance of each layer in the network varies, global pruning was employed.

In Table 2, the frames per second (FPS) were tested on the CPU with a batch size of 4. Various lightweight models were pruned using the L1, slimming (slim) [27], and lamp pruning methods. The results indicate that Lamp1.5 and L1-1.5 achieve the highest map scores. However, Lamp2.0 can achieve a lower parameter count and model size with a slight sacrifice in accuracy. Therefore, different distillation methods, namely CWD, masked intermediate map imitation constraint dissillation (MIMIC) [28], and mask generative dissillation (MGD), were applied to the Lamp2.0 model to balance speed and accuracy. The GH-YOLOX’s S model was employed as the teacher model, and the n model served as the student model, primarily distilling layers 16, 19, and 22 of the network, as illustrated in Figure 1. The experimental results demonstrate that adopting the CWD distillation method yields the highest map score of 90.1%.

**Figure 8:**
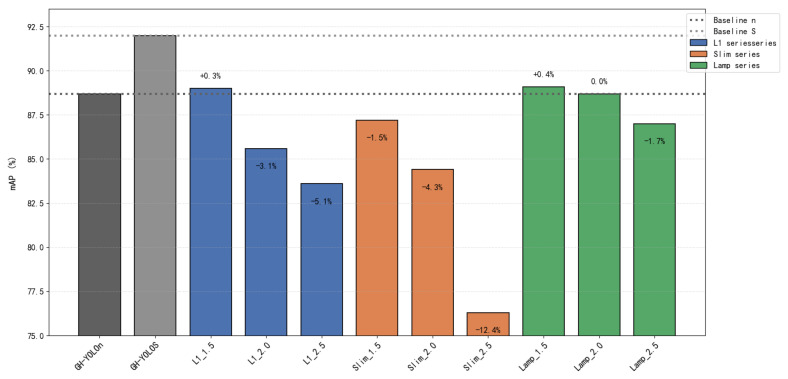
Performance comparison analysis of pruning methods.

As shown in Figure 8, when evaluating the performance (mAP) of various pruning methods, it is evident that the mAP of models processed via different pruning techniques generally demonstrates a downward trend. However, as the compression ratio of the lamp pruning method increases, the variation range of the mAP value remains the smallest among all methods. Specifically, L1-1.5 and Lamp-1.5 show mAP increases of 0.3% and 0.4%, respectively. In contrast, Lamp-2.0 maintains a stable mAP with no change, while Slim-2.5 experiences a significant mAP drop of 12.4% under a 2.5x compression ratio. This substantial compression in the latter case clearly undermines the model’s feature extraction capability. To date, considering the balance between compression efficiency and model performance, the lamp pruning method stands out as the optimal choice.

**Figure 9:**
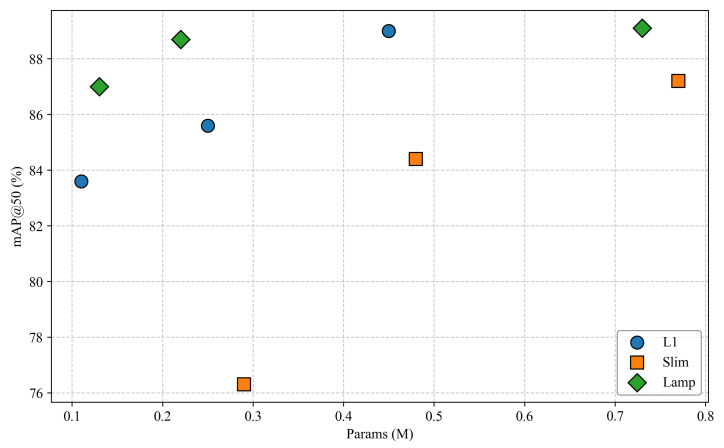
Pruning methods and parameters.

Figure 9 shows that the different pruning rates or the same pruning rate have different effects on the number of model parameters. Even if the pruning rate is the same, different methods have different degrees of compression on the number of model parameters. From the perspective of the change range, the model parameters of various methods decreased more under the compression ratio of 1.5× to 2.0× and 2.0× to 2.5×, and lamp could best take into account the number of parameters and model mAP. So Lamp-1.5 and Lamp-2.0 are the best choices in terms of the number of parameters and mAP.

**Figure 10:**
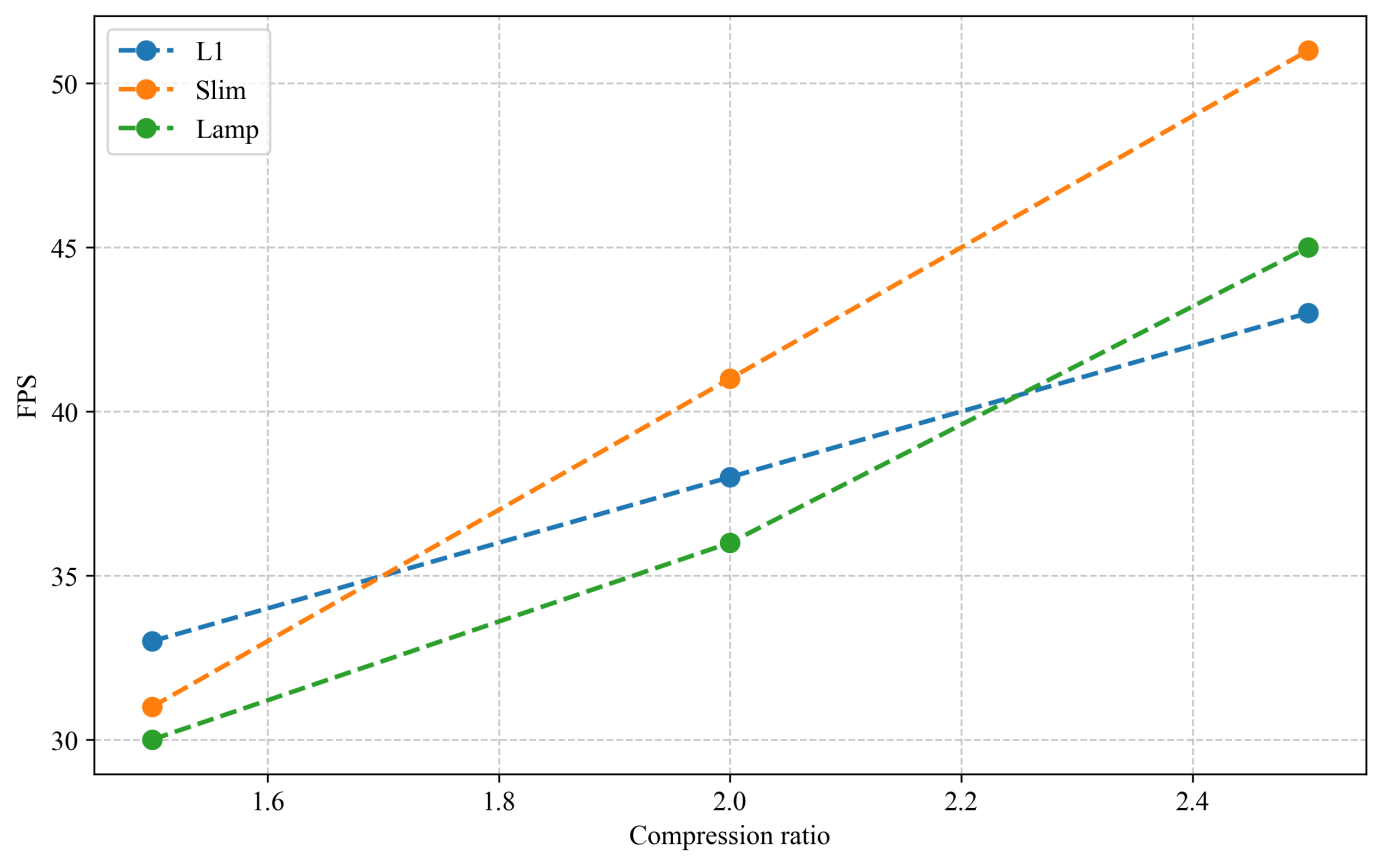
Compression ratio vs. inference speed.

As shown in Figure 10, the relationship between the compression ratio and inference speed is depicted. Generally, the FPS of all methods improves with an increasing compression ratio, but the growth rate diminishes. Although slim achieves higher speeds, especially at 2.0× and 2.5× compression ratios, where its FPS surpass those of the other two methods, its mAP declines more dramatically, particularly at the 2.5× compression ratio. Despite the significant speed increase, the mAP drops faster. In comparison, the FPS of Lamp-2.0 were slightly lower than those of L1-2.0, yet the mAP of Lamp-2.0 remained the same. Although Lamp-2.0’s speed is not the fastest, it still achieves 36 FPS, meeting the requirements for industrial real-time detection.

Table 3 shows that, under the same compression ratio, LAMP achieves a better accuracy–efficiency balance than the L1/slim method. For example, the mAP of LAMP-2.0 only decreases by 0.3% (88.7→88.4) when compressed by 2.0×, while the number of parameters decreases by 88% (1.1 M→0.22 M). The mAP of Slim-2.5 decreases by 12.4% when compressed by 2.5×, indicating that excessive compression will destroy the feature extraction ability, which needs to be repaired through subsequent distillation. Considered comprehensively, this section continues to compare different distillation methods with Lamp-2.0.

The S-model of GH-YOLOX is used as a teacher model, while the N-model is used as a student model, mainly distilling layers 16, 19, and 22 of the network. The experimental results are shown in Table 4: On the basis of Lamp-2.0, CWD (channelwise knowledge distillation) improves the mAP by 1.3% to 90.1%, surpassing the original teacher model (92.0% of S model → 90.1% of student model). It is proven that a channel-level knowledge transfer can effectively restore the feature expression ability of pruning loss compared with MIMIC and MGD [29] distillation.

## 7. Performance Comparison of Different Models and Dataset

To validate the effectiveness of the proposed GH-YOLOx, we compared it with five other models in terms of the Params size, model size, and mAP%. Additionally, to verify the model’s generalization ability, we conducted experiments on the fabric Data1 [5] and steel Data2 [6] datasets. As shown in Table 5, our lightweight model “n” outperforms other lightweight models such as YOLOv6, YOLOv5_Lite-e, and NanoDet-m [30] in terms of both accuracy and model size, as well as computational complexity. For the “S” model, it achieves the highest mAP, as shown in Table 5. Furthermore, by applying lamp pruning to the “n” model, we were able to improve the model’s accuracy to some extent while reducing the model’s parameter count. Finally, by distilling the pruned “n” model using CWD, we further enhanced the model’s detection capability. The final model achieves an accuracy of 90.1% and 83.2%, with a model size of 0.7, and a parameter count of 0.22. As shown in Table 5, these experimental results demonstrate the effectiveness of our lightweight network and the final lightweight framework.

### Detection Result

**Figure 11:**
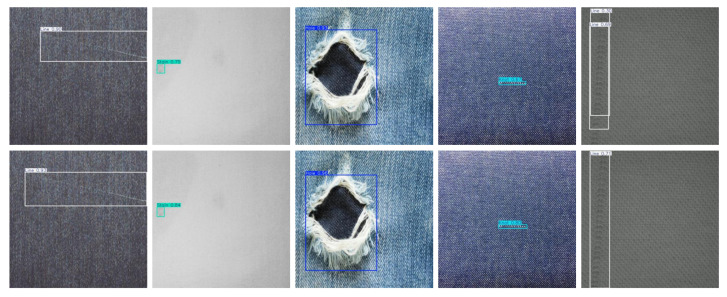
Visualized results of fabric-defect detection.

**Figure 12:**
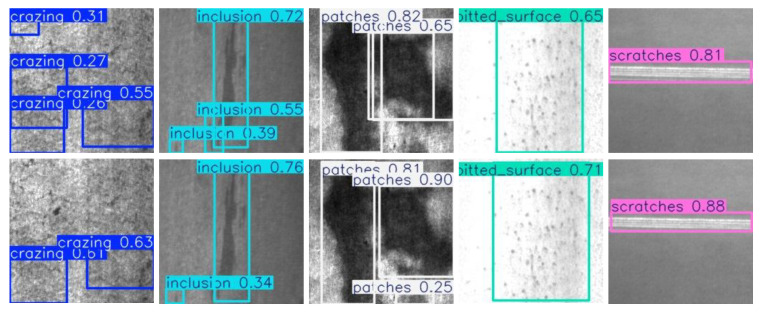
Visualization results of steel-defect detection.

According to the detection results of the improved model shown in Figure 11 and Figure 12, this model demonstrates a significant enhancement in detecting various types of defects, including line defects, stains, holes, nodules, rolled scales, inclusions, cracks, and pockmarked surfaces. Despite being lightweight, the detection capability of the model has not declined; instead, the detection accuracy and recall rate have both improved. This model particularly shows higher recognition accuracy and robustness in scenarios with small sample sizes and complex backgrounds. This further validates that the improved model can maintain or even enhance the performance of industrial defect detection while ensuring efficiency, demonstrating extensive application potential.

## 8. Conclusions

To address the issues of model complexity and computational resource consumption while fully considering the characteristics of fabric defect detection, this paper has proposed a comprehensive lightweighting solution. This solution combines lightweight ghost convolutions and the lightweight network GHNetV2, improving the backbone network and introducing dynamic convolutions and feature fusion modules to enhance attention to minor defects and feature expression capabilities. By adopting a lightweight shared convolution head, the multi-scale problem is addressed, and model compression is further achieved through pruning techniques, enhancing inference speeds. Finally, knowledge distillation is employed to enhance the accuracy of the pruned model. The improved lightweight network framework outperforms other mainstream lightweight models in terms of fewer parameters and a faster speed, and embedding various modules into other models also reduces the parameter count and improves performance. Moreover, pruning the lightweight network further compresses model size and boosts model speed, with a subsequent enhancement of model capabilities. This comprehensive lightweight network solution provides a feasible approach to the real-time deployment of fabric-defect detection. Furthermore, experimental results with steel defects demonstrate that, compared to other lightweight network models, the proposed method can further improve model accuracy while reducing model parameters. In the next steps, we will continue researching lightweighting methods to further improve model accuracy and reduce computational costs.

## Figures and Tables

**Table 1:** Model performance comparison.

Model	Configuration	mAP@0.5 (%)	FPS (ms)	Params (M)
YOLOv5	Baseline	88.3	4.7	2.5
	+A	89.2	4.0	2.0
	+B	89.4	4.9	2.2
	+C	88.9	3.5	1.8
	A+B+C	88.7	3.5	1.1
YOLOv8	Baseline	87.8	4.0	3.2
	+A	88.6	3.9	2.3
	+B	88.2	4.3	2.5
	+C	89.1	3.8	2.3
	A+B+C	89.1	3.4	1.6
GH-YOLOn	A+B+C	89.1	3.4	1.6

**Table 2:** Pruning.

Model	Model Size (MB)	FPS (S)	Params (M)	mAP@0.5 (%)
Our(A+B+C)n	2.7	25	1.1	88.7
Our(A+B+C)S	9.9	15	4.7	92.0
L1_1.5	1.2 (−1.5)	33 (+8)	0.45 (−0.65)	89.0 (+0.3)
L1_2.0	0.8 (−1.9)	38 (+13)	0.25 (−0.85)	85.6 (−3.1)
L1_2.5	0.5 (−2.2)	43 (+18)	0.11 (−0.99)	83.6 (−5.1)
Slim_1.5	1.9 (−0.8)	31 (+6)	0.77 (−0.33)	87.2 (−1.5)
Slim_2.0	1.3 (−1.4)	41 (+16)	0.48 (−0.62)	84.4 (−4.3)
Slim_2.5	0.9 (−1.8)	51 (+26)	0.29 (−0.81)	76.3 (−12.4)
Lamp_1.5	1.5 (−1.2)	30 (+5)	0.73 (−0.37)	89.1 (+0.4)
Lamp_2.0	0.7 (−2.0)	36 (+11)	0.22 (−0.88)	88.7 (+0.0)
Lamp_2.5	0.6 (−2.1)	45 (+20)	0.13 (−0.97)	87.0 (−1.7)

**Table 3:** Comparison of pruning methods.

Metric\Method	L1_1.5	Lamp_1.5	Lamp_2.0	Slim_2.5
Model compression ratio	1.5×	1.5×	2.0×	2.5×
mAP change	+0.3%	+0.4%	+0.0%	−12.4%
FPS improvement	+8	+5	+11	+26

**Table 4:** Knowledge distillation method comparison.

Method	mAP@0.5 (%)
CWD distillation	90.1 (+1.3%)
MIMIC distillation	88.9 (+0.2%)
MGD distillation	88.4 (−0.3%)

**Table 5:** Different models and dataset.

Model	Params Size (M)	Model Size (Mb)	mAP@0.5 (%)	
			Data1	Data2
YOLOv5	2.5	5.3	88.3	80.3
YOLOv6	4.2	8.7	83.5	80.0
YOLOv8	3.2	6.3	87.8	81.0
YOLOv5_lite-e	0.78	1.7	34.0	30.0
NanoDet-m	0.95	1.8	38.2	34.2
Our	0.22	0.7	90.1	83.2

## Data Availability

The links to the fabric dataset and neu surface defect dataset used in the paper are https://universe.roboflow.com/capstone-project-baym7/bkdn-huylv-datn-box (accessed on 20 June 2024) and http://faculty.neu.edu.cn/songkechen/zh_CN/zdylm/263270/list/index.htm (accessed on 20 June 2024). If you are interested in the Four-Fabric-Defects Dataset used in this paper, please send an email to 2215363086@mail.wtu.edu.cn and state the purpose.
